# Young patients show poor efficacy for immune checkpoint inhibitor combined therapy in metastatic gastrointestinal cancers

**DOI:** 10.3389/fonc.2023.1155019

**Published:** 2023-05-03

**Authors:** Yingnan Wang, Shasha Zhang, Fengbin Zhang, Lei Wang, Chensi Wu, Xiaoyun Zhang, Ruixing Zhang, Zhanjun Guo

**Affiliations:** ^1^ Department of Gastroenterology and Hepatology, The Fourth Hospital of Hebei Medical University, Shijiazhuang, China; ^2^ Department of Rheumatology, The Fourth Hospital of Hebei Medical University, Shijiazhuang, China; ^3^ Department of Thoracic Surgery, The Fourth Hospital of Hebei Medical University, Shijiazhuang, China

**Keywords:** gastrointestinal cancers, ICIs combined therapy, young, prognosis, irAEs

## Abstract

**Background:**

The impact of age on the efficacy and safety of immunotherapy remains controversial. The previous studies simply classified patients into younger and older groups, which might not reflect the real impact of young age on immunotherapy efficacy. The current study aimed to explore the efficacy and safety of immune checkpoint inhibitor (ICI) combined therapy in young (aged 18–44 years), middle-aged (aged 45–65 years), and old (aged >65 years) patients with metastatic gastrointestinal cancers (GICs), and further determine the role of immunotherapy in young patients.

**Methods:**

Patients with metastatic GIC including esophageal cancer (EC), gastric cancer (GC), hepatocellular cancer (HCC), and biliary tract cancer (BTC) who received ICI combination therapy were enrolled, divided into young (aged 18–44 years), middle-aged (aged 45–65 years), and old (aged >65 years) groups. The clinical characteristics, objective response rate (ORR), disease control rate (DCR), progression-free survival (PFS), overall survival (OS), and immune-related adverse events (irAEs) were compared among three groups.

**Results:**

A total of 254 patients were finally included, with 18, 139, and 97 cases in the young (aged 18–44 years), middle-aged (aged 45–65 years), and old (aged >65 years) groups, respectively. Compared to middle-aged and old patients, young patients had lower DCR (all *p* < 0.05) and also had inferior PFS (*p* < 0.001) and OS (*p =* 0.017). The multivariate analyses showed that young age was an independent prognostic factor for PFS [hazard ratio (HR) 3.474, 95% confidence interval (CI) 1.962–6.150, *p* < 0.001] and OS (HR 2.740, 95% CI 1.348–5.570, *p* = 0.005). Subsequent safety analyses referring to irAEs demonstrated no significant differences for distribution frequency among each age group (all *p* > 0.05), whereas patients with irAEs displayed better DCR (*p* = 0.035) and PFS (*p* = 0.037).

**Conclusion:**

Younger GIC patients (aged 18–44 years) showed poor efficacy for ICI combined therapy, and irAEs could be used as a clinical biomarker to predict ICI efficacy in metastatic GIC patients.

## Introduction

Gastrointestinal cancers (GIC), mostly composed of esophageal cancer (EC), gastric cancer (GC), colorectal cancer (CRC), pancreatic cancer (PC), hepatocellular cancer (HCC), and biliary tract cancer (BTC), account for 26% of global cancer incidence and 35% of all cancer-related deaths, which makes them one of the most common groups of malignancy worldwide (1). A large portion of patients have unresectable or metastatic disease at the time of diagnosis due to the late detection and high heterogeneity of these malignancies. Traditional chemotherapy, radiotherapy, and targeted therapy have been disappointing with a dismal 5-year survival in advanced stage disease (2). Over the last few decades, immune checkpoint inhibitors (ICIs) against programmed death receptor 1 (PD-1), programmed death receptor ligand 1 (PD-L1), and cytotoxic T lymphocyte-associated antigen-4 (CTLA-4) have emerged as a revolutionary option for cancer treatment. However, unlike in lung cancer or melanoma, the response rates to immunotherapy in GIC are relatively low (3). More recently, ICIs plus other therapies like other ICIs, chemotherapy, radiotherapy, and targeted therapy have been shown to synergistically promote the efficacy of ICI monotherapy in GIC patients, which has been confirmed in several randomized controlled trials (RCTs) and systematic reviews (4–6). However, there is a lack of knowledge about predicting response to combining immunotherapies of GIC patients.

Currently, cancer is classically understood as a “disease of aging”, with a significantly higher incidence in patients aged ≥65 years (7). Compared with younger people, the immune system of older people can undergo a remodeling process during aging, which involves immune dysregulation in both cellular and humoral responses (8). It is speculated that older patients seem to obtain limited benefits from ICI treatment compared to younger patients. Some reports have demonstrated that ICI therapy is less effective in older people than in younger people (9, 10). However, a recent study found that young patients showed a worse response to immunotherapy than old patients. A further study reported that regulatory T cells (Tregs) are specifically increased within the tumor microenvironment of young patients. CD8+ T cells, which are the primarily activated cell type by anti-PD-1 checkpoint targeting as a tumor cell killer, are also decreased in melanoma tumors from younger patients (11). Meanwhile, many studies did not find ICI efficacy difference between young and elderly patients (12–14). Obviously, the definitions of young patients were quite inconsistent in previous studies and most studies simply classified patients into younger and older groups, which may not reflect the real impact of young age on immunotherapy efficacy. Cheng et al. indicated that age-specific impact on GC survival was possibly in a V-shaped distribution, i.e., the middle-aged subgroups showed better prognosis than the younger and oldest subgroups (15). However, it is not clear whether age will affect the immunotherapy efficacy like this pattern.

Therefore, in the present study, we divided GIC patients into three subgroups according to age of diagnosis: young (aged 18–44 years), middle-aged (aged 45–65 years), and old (aged >65 years), asutilized by previous studies (16–18), to compare the immunotherapy efficacy and safety of young age with that of middle age and old age, respectively, and hoped to further determine the role of immunotherapy in young GIC patients.

## Methods

### Patient screening and data collection

This study retrospectively analyzed the medical data of patients with EC, GC, HCC, and BTC who received ICI combination therapy in the Fourth Hospital of Hebei Medical University from November 2018 to December 2021. Inclusion criteria were as follows (1): imaging confirmed as metastatic GIC before treatment initiation; (2) patients who received ICI combination therapy; (3) detailed and complete clinical data; and (4) no relevant infection or acute or chronic inflammatory reaction before treatment initiation. The metastatic patterns were defined as any metastatic lesion in the liver, lung, bone, peritoneum, brain, distant lymph nodes, and other distant organs. Exclusion criteria were as follows: (1) patients treated with anti-PD-1 monotherapy; (2) combined with other tumor history, infection, or blood system disease; (3) patients who gave up treatment or refused to accept assessment; and (4) patients without complete medical records and laboratory results.

Study characteristics were extracted, including age, sex, performance status (PS) score, cancer type, therapy line, treatment modality, and immune-related adverse events (irAEs). Peripheral venous blood was collected from all patients on an empty stomach within 1 week before treatment initiation. The following laboratory indexes were collected: albumin level, neutrophil-to-lymphocyte ratio (NLR), lactate dehydrogenase (LDH), and creatinine clearance rate (Ccr). The study protocol was tested and approved by the ethics committee of The Fourth Hospital of Hebei Medical University (2020KS001), and due to the retrospective nature of this study, a waiver of informed consent was applied for these analyses.

### Treatment and assessment

Patients received ICI combination with chemotherapy or/and targeted therapy every 3 weeks in the clinical setting. Treatment continued until disease progression, clinical worsening, treatment-related adverse events, or patient refusal. **Table S1** lists cancer type, treatment lines, number of patients in each therapeutic schedule, and types of immunotherapy drugs, targeted drugs, and chemotherapy drugs. Before starting the treatments, all patients received enhanced thoracic–abdominal–pelvic computed tomography (CT) scan. The CT image sets were retrieved and re-assessed according to the staging criteria for GIC, including EC, GC, HCC, and BTC. Treatment response was assessed with the Response Evaluation Criteria in Solid Tumours (RECIST) version 1.1 criteria (https://ctep.cancer.gov/protocolDevelopment/docs/recist_guideline.pdf#search=%22RECIST%201.1%22) and the modified RECIST 1.1 for immune-based therapeutic (iRECIST). Complete response (CR) was defined as the complete disappearance of the target lesion after chemotherapy. Partial response (PR) was defined as a reduction in the total diameter of each target lesion by 30% or more. Progressive disease (PD) was defined as at least a 20% increase in the sum of the long diameters of all target lesions and an absolute value for the increase of more than 5 mm, or the appearance of new lesions. Stable disease (SD) was defined as no change in target lesions. The objective response rate (ORR) was defined as the percentage of patients with a CR or PR among all the treated patients. The disease control rate (DCR) was defined as the percentage of patients who have achieved CR, PR, and SD. The toxicities of anti-PD-1 therapy were assessed according to the National Cancer Institute Common Terminology Criteria for Adverse Event (NCI-CTCAE), version per protocol (https://ctep.cancer.gov/protocolDevelopment/electronic_applications/ctc.htm#ctc_40). Other adverse events including progression of malignancy and adverse events caused by chemotherapy drugs or targeted drugs were excluded.

### Follow-up and study outcomes

Clinical information was obtained from patients’ medical records. All patients were followed up *via* re-hospitalization or re-examinations in the outpatient clinic or by telephone until mortality due to any reasons or loss of follow-up. Progression-free survival (PFS) was defined as the period from treatment initiation until the date of disease progression, death, or study cutoff, whichever occurred first. Overall survival (OS) was defined as time from treatment initiation to the date of death from any cause or study cutoff. The end point of follow-up was 1 May 2022 or the date of death.

### Statistical analysis

All statistical analyses were performed using SPSS 24.0 software package (SPSS Inc., Chicago, IL, USA) and GraphPad Prism 9 (GraphPad Software, San Diego, CA). Qualitative variables and continuous variables were described as frequencies, percentages, mean, standard deviation, and median. Group comparison of qualitative variables was performed using Pearson’s chi-squared test or two-sided Fisher’s exact test, while continuous variables were compared with Student’s *t*-tests or Mann–Whitney *U*-test. Survival among different age groups was evaluated using the Kaplan–Meier method and log-rank tests. Univariate and multivariate Cox proportional hazards were performed to explore prognostic factors for survival. *p* < 0.05 was considered statistically significant, and all reported *p*-values are two-sided.

## Results

### The baseline clinical characteristics of patients

**Figure 1:**
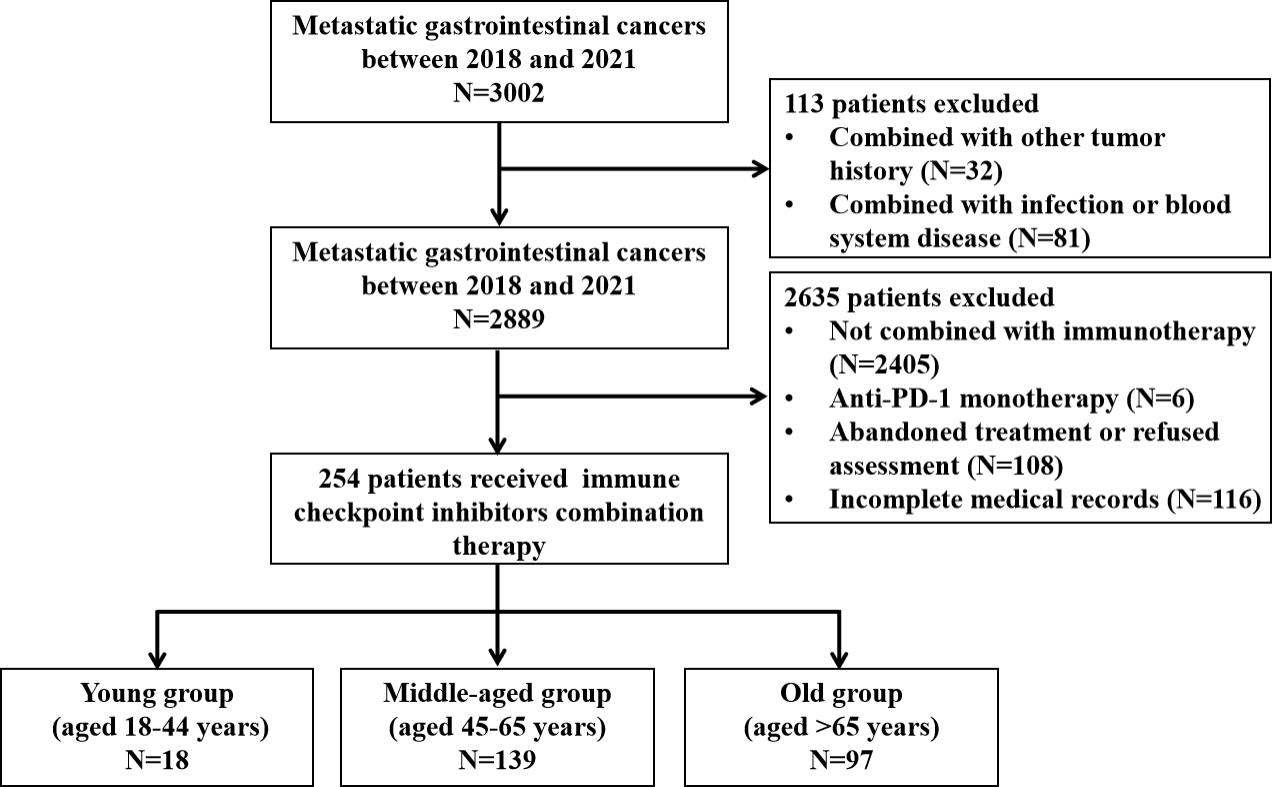
Flowchart of patient enrollment and exclusion.

A total of 254 patients were enrolled in this study, with 18 (7.1%), 139 (54.7%), and 97 (38.2%) cases in the young (aged 18–44 years), middle-aged (aged 45–65 years), and old (aged >65 years) groups, respectively. **Figure 1** shows the selection procedure of the study cohort based on inclusion and exclusion criteria. The median age of the three groups was 39 (range, 22–44 years), 58 (range, 45–65 years), and 70 (range, 66–88 years) years. Among them, there were 79 esophageal cancer patients who received anti-PD-1 antibodies in combination with chemotherapy or/and targeted therapy, 106 gastric cancer patients who received anti-PD-1 antibodies plus chemotherapy or/and targeted therapy, 51 hepatocellular cancer patients who received anti-PD-1 antibodies in combination with targeted therapy, and 18 biliary tract cancers patients who received anti-PD-1 antibodies in combination with targeted therapy or/and chemotherapy (**Table S1**).

The detailed baseline clinical characteristics of different age groups are summarized in **Table 1**. Compared with the middle-aged and old groups, the proportions of ICI plus chemotherapy were significantly lower in the young group (all *p* < 0.05). Young patients also have higher baseline Ccr level than those of middle-aged and old patients (all *p* < 0.05). Meanwhile, young patients were different from the middle-aged group in the proportion of female patients (*p* = 0.036). However, the young group shared some features with the middle-aged group rather than with the old group, including the proportion of esophageal cancer (*p* = 0.008), ICI plus targeted therapy (*p* = 0.021), and the baseline LDH level (*p* = 0.016).

**Table 1:** The baseline clinical characteristics of different age groups.

Characteristic	Young (Y, n=18)	Middle-aged (M, n=139)	Old (O, n=97)	*P*-value (Y vs. M)	*P*-value (Y vs. O)
Gender					
Male	10 (55.6%)	112 (80.6%)	67 (69.1%)	0.036	0.263
Female	8 (44.4%)	27 (19.4%)	30 (30.9%)		
PS score					
≤ 1	11 (61.1%)	79 (56.8%)	44 (57.1%)	0.730	0.219
> 1	7 (38.9%)	60 (43.2%)	53 (42.9%)		
Cancer type					
Esophageal cancer	1 (5.5%)	42 (30.2%)	36 (37.1%)	0.054	0.008
Gastric cancer	9 (50.0%)	52 (37.4%)	45 (46.4%)	0.302	0.778
Hepatocellular cancer	5 (27.8%)	37 (26.6%)	9 (9.3%)	1.000	0.070
Biliary tract cancers	3 (16.7%)	8 (5.8%)	7 (7.2%)	0.224	0.395
Therapy line					
≤ 2	12 (66.7%)	113 (81.3%)	80 (82.5%)	0.255	0.223
≥ 3	6 (33.3%)	26 (18.7%)	17 (17.5%)		
Treatment modality					
ICI plus chemotherapy	4 (22.2%)	65 (46.8%)	55 (56.7%)	0.048	0.007
ICI plus targeted therapy	10 (55.6%)	57 (41.0%)	27 (27.8%)	0.240	0.021
ICI plus chemotherapy and targeted therapy	4 (22.2%)	17 (12.2%)	15 (15.5%)	0.421	0.716
Albumin (g/L)	37.4±6.0	40.6±24.1	38.2±4.3	0.576	0.515
NLR	5.3±3.4	4.3±2.9	4.4±3.4	0.214	0.315
LDH (U/L)	333.8±204.9	232.6±121.1	203.2±86.0	0.055	0.016
Ccr (ml/min)	136.9±33.4	110.8±29.3	84.7±22.0	0.005	<0.001

PS, performance status; ICI, immune checkpoint inhibitor; NLR, neutrophil-to-lymphocyte ratio; LDH, lactate dehydrogenase; Ccr, creatinine clearance rate.

### Treatment efficacy

The patients with PD were all patients with icPD (immunity confirmed PD). As shown in **Table 2**, the ORR of the three age groups was 11.1% (2/18), 26.6% (37/139), and 26.8% (26/97), with no significant difference for each age group (all *p* > 0.05). Stratified by therapy line, ICI combined therapy in the first-line or second-line setting was associated with higher ORR than that in later line treatment (*p* = 0.044). The DCR of the three age groups was 38.9% (7/18), 74.1% (103/139), and 74.2% (72/97), respectively. We did not find a significant difference between the middle-aged and old groups (*p* = 0.913), but the young group showed a significantly lower DCR (all *p* < 0.05). We also observed that the patients with good PS, esophageal cancer, ICI combined therapy in the first-line or second-line setting, an occurrence of irAEs, and baseline albumin level > 39.2 g/L displayed higher DCR (all *p* < 0.05). Moreover, ICI plus chemotherapy had higher DCR than ICI combined with other therapies (*p* = 0.003).

**Table 2:** The clinical response to treatment according to characteristics of patients.

Characteristic	ORR (*N* = 65)		*p*-value ^b^	DCR (*N* = 182)		*p*-value ^b^
	Events (%)	95% CI ^a^		Events (%)	95% CI ^a^	
Age (years)						
18–44	2 (11.1)	5.0%–27.2%	0.253	7 (38.9)	13.9%–63.8%	0.002
45–65	37 (26.6)	19.2%–34.1%	0.975	103 (74.1)	66.7%–81.5%	0.913
> 65	26 (26.8)	17.8%–35.8%	0.260	72 (74.2)	65.4%–83.1%	0.004
Gender						
Male	52 (27.5)	21.1%–33.9%	0.231	134 (70.9)	64.4%–77.4%	0.649
Female	13 (20.0)	10.0%–30.0%		48 (73.8)	62.9%–84.8%	
PS score						
≤1	38 (28.4)	20.6%–36.1%	0.285	109 (81.3)	74.7%–88.0%	0.000
>1	27 (22.5)	14.9%–30.1%		73 (60.8)	52.0%–69.7%	
Cancer type						
Esophageal cancer	26 (32.9)	22.3%–43.5%	0.072	65 (82.3)	73.7%–90.9%	0.010
Gastric cancer	26 (24.5)	16.2%–32.9%	0.743	71 (67.0)	57.9%–76.1%	0.191
Hepatocellular cancer	8 (15.7)	5.4%–26.0%	0.070	33 (64.7)	51.1%–78.3%	0.218
Biliary tract cancers	5 (27.8)	4.9%–50.7%	1.000	13 (72.2)	49.3%–95.1%	0.956
Therapy line						
≤2	58 (28.3)	22.1%–34.5%	0.044	158 (77.1)	71.3%–82.9%	0.000
≥3	7 (14.3)	4.1%–24.4%		24 (49.0)	34.5%–63.5%	
Treatment modality						
ICI plus chemotherapy	36 (29.0)	20.9%–37.1%	0.220	99 (79.8)	72.7%–87.0%	0.003
ICI plus targeted therapy	19 (20.2)	11.9%–28.5%	0.132	58 (61.7)	51.7%–71.7%	0.411
ICI plus chemotherapy and targeted therapy	10 (27.8)	12.4%–43.1%	0.745	25 (69.4)	53.6%–85.3%	0.189
IrAEs						
No	41 (24.8)	18.2%–31.5%	0.712	111 (67.3)	60.0%–74.5%	0.035
Yes	24 (27.0)	17.6%–36.4%		71 (79.8)	71.3%–88.5%	
Albumin (g/L) ^c^						
≤39.2	29 (22.3)	15.1%–29.6%	0.220	86 (66.2)	57.9%–74.4%	0.046
>39.2	36 (29.0)	20.9%–37.1%		96 (77.4)	70.0%–84.9%	
NLR ^c^						
≤3.6	33 (25.2)	14.0%–28.5%	0.880	92 (70.2)	67.1%–82.5%	0.603
>3.6	32 (26.0)	21.8%–38.0%		90 (73.2)	60.3%–76.7%	
LDH (U/L) ^c^						
≤187.5	27 (21.3)	17.7%–32.7%	0.114	95 (74.8)	62.3%–78.2%	0.265
>187.5	38 (29.9)	18.2%–33.9%		87 (68.5)	65.2%–81.1%	
Ccr (ml/min) ^c^						
≤99.5	36 (27.7)	19.9%–35.5%	0.432	96 (73.8)	66.2%–81.5%	0.427
>99.5	29 (23.4)	15.8%–30.9%		86 (69.4)	61.1%–77.6%	

ORR, objective response rate; DCR, disease control rate; CI, confidence interval; PS, performance status; ICI, immune checkpoint inhibitor; irAEs, immune-related adverse events; NLR, neutrophil-to-lymphocyte ratio; LDH, lactate dehydrogenase; Ccr, creatinine clearance rate.

^a^Exact Clopper-Pearson 95% confidence interval, the true probability of ORR or DCR falls within the interval with 95% probability; ^b^Chi-square test, or exact chi-square test if any expected cell size <5; ^c^Take the median of this cohort as cutoff value.

### Prognostic analysis

**Figure 2:**
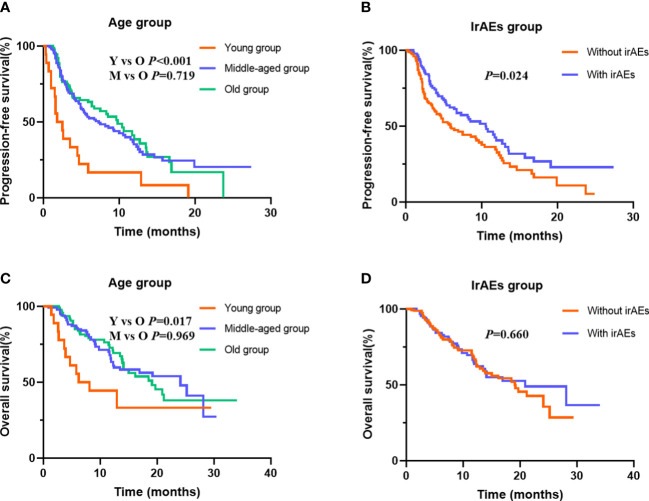
Kaplan–Meier curves for progression-free survival (PFS) and overall survival (OS) stratified by age and immune-related adverse events (irAEs). **(A)** for PFS in different age group; **(B)** for PFS in patients with or without irAEs; **(C)** for OS in different age group; **(D)** for OS in patients with or without irAEs.

To determine the potential role of age on PFS, Kaplan–Meier plots were applied. The median PFS was 1.8, 7.3, and 9.8 months in the young, middle-aged, and old groups. We did not find significant PFS difference between the middle-aged and old groups (*p* = 0.719), but young patients had the worst PFS (*p* < 0.001) (**Figure 2A**). Stratified by PS score and therapy line, we found that the patients with good PS and who received ICI combination therapy in the first-line or second-line setting were associated with better PFS (all *p* < 0.05). Moreover, we also observed an improvement in terms of PFS in patients who developed irAEs (*p* = 0.024) (**Figure 2B**). Next, we performed multivariate analyses to identify independent prognostic factors for PFS. As the Cox-proportional hazard model in **Table 3** shows, age was an independent prognostic factor for PFS, with the hazard ratio (HR) of 3.474 [95% confidence interval (CI) 1.962–6.150, *p* < 0.001] for those aged <45 years and 1.137 (95% CI 0.798–1.621, *p* = 0.478) for 45–65 years. Other independent prognostic factors for PFS were PS score (HR 1.605, 95% CI 1.151–2.240, *p* = 0.005), therapy line (HR 2.092, 95% CI 1.450–3.019, *p* < 0.001), and irAEs (HR 0.694, 95% CI 0.492–0.978, *p* = 0.037).

**Table 3:** Univariate and multivariate analyses for progression-free survival.

Characteristic	Univariate analysis		Multivariate analysis		
	HR (95% CI)	*p*-value	*B*	HR (95% CI)	*p*-value
Age (years)					
>65	Reference	<0.001		Reference	<0.001
45–65	1.066 (0.753–1.510)	0.719	0.128	1.137 (0.798–1.621)	0.478
18–44	2.896 (1.672–5.017)	<0.001	1.245	3.474 (1.962–6.150)	<0.001
Gender					
Male	Reference	0.392			
Female	1.166 (0.819–1.661)				
PS score					
≤1	Reference	0.048		Reference	0.005
>1	1.378 (1.000–1.898)		0.473	1.605 (1.151–2.240)	
Cancer type					
Esophageal cancer	Reference	0.515			
Gastric cancer	1.276 (0.872–1.868)	0.209			
Hepatocellular cancer	0.947 (0.590–1.519)	0.820			
Biliary tract cancers	1.076 (0.584–1.981)	0.815			
Therapy line					
≤2	Reference	<0.001		Reference	<0.001
≥3	2.109 (1.466–3.035)		0.738	2.092 (1.450–3.019)	
Treatment modality					
ICI plus chemotherapy	Reference	0.348			
ICI plus targeted therapy	1.105 (0.783–1.560)	0.569			
ICI plus chemotherapy and targeted therapy	1.461 (0.874–2.441)	0.148			
IrAEs					
No	Reference	0.024		Reference	0.037
Yes	0.682 (0.488–0.954)		−0.365	0.694 (0.492–0.978)	
Albumin (g/L) ^a^					
≤39.2	Reference	0.268			
>39.2	0.834 (0.606–1.150)				
NLR ^a^					
≤3.6	Reference	0.586			
>3.6	1.093 (0.794–1.504)				
LDH (U/L) ^a^					
≤187.5	Reference	0.686			
>187.5	0.936 (0.679–1.289)				
Ccr (ml/min) ^a^					
≤99.5	Reference	0.073			
>99.5	1.341 (0.972–1.850)				

PS, performance status; ICI, immune checkpoint inhibitor; irAEs, immune-related adverse events; NLR, neutrophil-to-lymphocyte ratio; LDH, lactate dehydrogenase; Ccr, creatinine clearance rate; B, beta coefficient; HR, hazard ratio; CI, confidence interval.

^a^ Take the median of this cohort as cutoff value.

At data cutoff, 99 (40.0%) deaths had occurred, 146 (57.5%) patients were alive, and 9 (3.5%) cases were lost to follow-up. The median OS of the three age groups was 6.2, 24.1, and 19.1 months, respectively. **Figure 2C** shows that old age was not inferior to middle age in predicting OS (*p* = 0.969), but the young age group had the worst OS (*p* = 0.017). In addition, **Table 4** shows that the patients with good PS, who received ICI combination therapy in the first-line or second-line setting and ICI plus chemotherapy, and with baseline albumin level > 39.2 g/L were associated with better OS (all *p* < 0.05). Moreover, patients with irAEs showed superior OS compared with patients without irAEs, but without statistical difference (*p* = 0.660) (**Figure 2D**). Multivariate Cox analysis indicated that young age was an independent prognostic factor for OS (HR 2.740, 95% CI 1.348–5.570, *p* = 0.005). Moreover, therapy line (HR 1.753, 95% CI 1.092–2.812, *p* = 0.020) and baseline albumin level (HR 0.457, 95% CI 0.292–0.714, *p* = 0.001) remained as independent prognostic factors for OS (**Table 4**).

**Table 4:** Univariate and multivariate analyses for overall survival.

Characteristic	Univariate analysis		Multivariate analysis		
	HR (95% CI)	*p*-value	*B*	HR (95% CI)	*p*-value
Age (years)					
>65	Reference	0.036		Reference	0.005
45–65	0.992 (0.649–1.514)	0.969	−0.112	0.894 (0.574–1.392)	0.619
18–44	2.277 (1.158–4.478)	0.017	1.008	2.740 (1.348–5.570)	0.005
Gender					
Male	Reference	0.101			
Female	1.420 (0.934–2.160)				
PS score					
≤1	Reference	0.009		Reference	0.271
>1	1.707 (1.145–2.545)		0.250	1.284 (0.823–2.005)	
Cancer type					
Esophageal cancer	Reference	0.338			
Gastric cancer	1.299 (0.791–2.134)	0.302			
Hepatocellular cancer	1.635 (0.964–2.771)	0.068			
Biliary tract cancers	1.229 (0.566–2.666)	0.602			
Therapy line					
≤2	Reference	0.015		Reference	0.020
≥3	1.762 (1.118–2.777)		0.561	1.753 (1.092–2.812)	
Treatment modality					
ICI plus chemotherapy	Reference	0.044		Reference	0.438
ICI plus targeted therapy	1.696 (1.120–2.567)	0.013	0.247	1.280 (0.813–2.015)	0.286
ICI plus chemotherapy and targeted therapy	1.333 (0.643–2.763)	0.440	−0.132	0.876 (0.413–1.857)	0.730
IrAEs					
No	Reference	0.660			
Yes	0.913 (0.607–1.372)				
Albumin (g/L) ^a^					
≤39.2	Reference	<0.001		Reference	0.001
>39.2	0.438 (0.290–0.661)		−0.784	0.457 (0.292–0.714)	
NLR ^a^					
≤3.6	Reference	0.168			
>3.6	1.320 (0.890–1.959)				
LDH (U/L) ^a^					
≤187.5	Reference	0.382			
>187.5	1.193 (0.804–1.770)				
Ccr (ml/min) ^a^					
≤99.5	Reference	0.538			
>99.5	1.132 (0.763–1.681)				

PS, performance status; ICI, immune checkpoint inhibitor; irAEs, immune-related adverse events; NLR, neutrophil-to-lymphocyte ratio; LDH, lactate dehydrogenase; Ccr, creatinine clearance rate; B, beta coefficient; HR, hazard ratio; CI, confidence interval.

^a^Take the median of this cohort as cutoff value.

### Safety analysis

The overall incidence of irAEs was 35.0% (89/254) with grade 3 or higher irAEs of 3.9% (10/254). The most common irAEs were thyroid disorders (50/89, 56.2%), skin disorders (16/89, 18.0%), and elevated cardiac enzymes (10/89, 11.2%). **Table 5** shows the relationship between characteristics of patients and irAEs. The results indicated no significant differences in the incidence of irAEs for each age group (all *p* > 0.05). IrAEs occurred in 25 patients with hepatocellular cancer (25/51, 49.0%), which was significantly higher than those with other GICs (*p* = 0.019). However, compared with other GICs, the incidence of irAEs in gastric cancer (25/106, 23.6%) is significantly lower (*p* = 0.001). Meanwhile, when irAEs were evaluated according to sex and treatment modality, women were more likely to experience irAEs than men (*p* = 0.029), and ICI plus chemotherapy significantly decreased the rate of irAEs than ICI combined with other therapies (*p* = 0.006).

**Table 5:** Relationship between characteristics of patients and immune-related adverse events.

Characteristic	With irAEs N (%)	Without irAEs N (%)	*p*-value ^a^
Age (years)			
18–44	5 (27.8)	13 (72.2)	0.361
45–65	54 (38.8)	85 (61.2)	0.211
>65	30 (30.9)	67 (69.1)	0.790
Gender			
Male	59 (31.2)	130 (68.8)	0.029
Female	30 (46.2)	35 (53.8)	
PS score			
≤1	44 (32.8)	90 (67.2)	0.437
>1	45 (37.5)	75 (62.5)	
Cancer type			
Esophageal cancer	29 (36.7)	50 (63.3)	0.708
Gastric cancer	25 (23.6)	81 (76.4)	0.001
Hepatocellular cancer	25 (49.0)	26 (51.0)	0.019
Biliary tract cancers	10 (55.6)	8 (44.4)	0.058
Therapy line			
≤2	76 (37.1)	129 (62.9)	0.165
≥3	13 (26.5)	36 (73.5)	
Treatment modality			
ICI plus chemotherapy	37 (29.8)	87 (70.2)	0.006
ICI plus targeted therapy	45 (47.9)	49 (52.1)	0.056
ICI plus chemotherapy and targeted therapy	7 (19.4)	19 (80.6)	0.767
Albumin (g/L) ^b^			
≤39.2	46 (35.4)	84 (64.6)	0.906
>39.2	43 (34.7)	81 (65.3)	
NLR ^b^			
≤3.6	44 (33.6)	87 (66.4)	0.617
>3.6	45 (36.6)	78 (63.4)	
LDH (U/L) ^b^			
≤187.5	39 (30.7)	88 (69.3)	0.148
>187.5	50 (39.4)	77 (60.6)	
Ccr (ml/min) ^b^			
≤99.5	44 (33.8)	86 (66.2)	0.683
>99.5	45 (36.3)	79 (63.7)	

irAEs, immune-related adverse events; PS, performance status; ICI, immune checkpoint inhibitor; NLR, neutrophil-to-lymphocyte ratio; LDH, lactate dehydrogenase; Ccr, creatinine clearance rate. ^a^Chi-square test, or exact chi-square test if any expected cell size <5; ^b^Take the median of this cohort as cutoff value.

## Discussion

Despite the fact that ICI immunotherapies have changed the landscape of cancer treatment for several advanced solid tumors, little is known about the impact of age on clinical efficacy and safety of ICI combined therapy in GIC patients. A meta-analysis performed indicated comparable efficacy of ICI-based combination therapy in younger and older patients with non-small cell lung cancer (NSCLC) with a cutoff age of 65 years (19). Another meta-analysis also did not find a difference in survival benefit of immunotherapy in older (≥65 years) *vs*. younger (<65 years) patients including 37 phase 2 or 3 randomized clinical trials of 23,760 patients (20). Patients were commonly classified into young and old groups based on the different cutoff value of age (65 or 70 years) (13, 19), which may partly lead to the failure of identifying the role of immunotherapy in young patients. However, we believe that the immune function of people of different ages is different, and the analysis of only older than or younger than an age point may mask the response of different age groups to ICIs. In the current study, we compared the clinical efficacy and safety of ICIs combined therapy of young age with that of middle age and old age. Patients aged 18–44 years showed a lower DCR and poorer survival, but there was no significant difference on the efficacy of ICI combination therapy between middle-aged (aged 45–65 years) and old (aged >65 years) patients, which indicated that young GIC patients showed poor efficacy for ICI combined therapy.

Several meta-analyses of clinical trials in multiple cancer types treated with ICI demonstrated similar results to our study and found that older patients can benefit more from immunotherapy than younger patients (21–24). However, the cause of low immunotherapy response in young patients is still unclear and several reasons may explain this. Castro et al. presented evidence that younger patients showed the strongest effects of major histocompatibility complex (MHC)-based driver mutation selection, which may influence the availability of mutant peptides capable of driving effective response to ICI therapy (25). A recent study by Kugel et al. indicated that intratumoral CD8+ T cells:Treg ratios of younger melanoma patients treated with PD-1 inhibitors significantly decreased compared to older patients and a similar result was observed in young mice (11). Therefore, young GIC patients may not be good candidates for immunotherapy, and further studies with evidence of high level are needed.

In addition, young patients have higher baseline LDH levels than old patients in our cohort. Elevated LDH is a negative prognostic biomarker not only because it is a key enzyme involved in cancer metabolism, but also because it alters the tumor microenvironment in hematologic and solid neoplasms, allowing tumor cells to suppress and evade the immune system (26). Its increase exhibited a negative effect for ipilimumab on metastatic melanoma in clinical applications (27, 28). However, we did not find elevated LDH to be associated with the immunotherapy efficacy and survival of patients in our study. The difference from other studies may be explained by cutoff value variations. Nonetheless, this might explain at least partly the poor ICI response in young patients in our study, and prospective new studies are needed to provide more information on this subject. Moreover, with the improvement of health habits, there are fewer and fewer opportunities to contact pathogenic microorganisms in childhood so as to obtain a sound immune system. Patients older than 45 years old may have much stronger immune systems to initiate immune response for this reason. Nevertheless, our results suggested that a detailed age stratification should be carried out when evaluating the efficacy of immunotherapy to deal with an aging society.

We also explored the safety of ICI combined therapy in young, middle-aged, and old patients. The overall incidence of irAE was 35.0% with grade 3 or higher irAEs of 3.9% in GIC, which was comparable with the previously reported incidence (29, 30). The most common adverse events were thyroid disorders, skin disorders, and elevated cardiac enzymes. Although the creatinine clearance rate significantly decreased with age, we found no significant differences in the incidence of irAEs for each age group. Several retrospective reviews focused on this issue also did not find statistically significant differences in irAEs based on age (31–33), so did another retrospective case–control study of patients involving melanoma, renal cell carcinoma, or NSCLC with three age groups: <65 years old, 65–74 years old, and ≥75 years old (34). These results suggest that ICIs could be safely used in older patients. Our study also observed significantly better DCR and a higher PFS in irAE patients, which confirmed the suggestions that irAE onset might become the first clinical biomarker for anti-PD-1 antibody response in patients with indications to receive ICIs (35–37). Our study also showed that women were more likely to develop irAEs than men, and ICI plus chemotherapy significantly decreased the rate of irAEs than ICI combined with other therapies. However, our series did not explore the relationships for different irAE symptoms and grades with different characteristics and immunotherapy response due to the relatively small sample size, and further research is necessary to explore this further.

The present study had some limitations, namely, its retrospective nature, patients having different treatment backgrounds, patients coming from one center, and the small sample size (*n* = 18) of those <45 years. However, to our knowledge, this was the first study that determined the role of ICI combined therapy in GIC patients with detailed age stratification. Findings from this pilot project would build the foundation for future research with more patients and more centers. Given this, there is an urgent need for a larger, multicenter prospective study to explore the predictive power of the age for ICI combined therapy to consolidate our findings.

## Conclusions

In summary, younger GIC patients (aged 18–44 years) showed poor efficacy for ICI combined therapy, and irAEs could be used as a clinical biomarker to predict ICI efficacy in GIC patients. Further studies are warranted to figure out the age-specific mechanisms of ICI combined therapy.

## Data availability statement

The original contributions presented in the study are included in the article/**Supplementary Material**. Further inquiries can be directed to the corresponding author.

## Ethics statement

All procedures were supervised and approved by the ethics committee of the Fourth Hospital of Hebei Medical University (2020KS001). Written informed consent for participation was not required for this study in accordance with the national legislation and the institutional requirements.

## Author contributions

YW designed the experiments, collected the data of patients, and wrote the paper. SZ, FZ and LW followed up the patients. CW, XZ and RZ performed statistical analysis and collected the data. ZG designed the experiments, conceived the concept, and wrote the paper. All authors contributed to the article and approved the submitted version.
