# Supplementary material for: Young patients show poor efficacy for immune checkpoint inhibitor combined therapy in metastatic gastrointestinal cancers
Source: Front Oncol. 2023 May 3;13:1155019. doi: 10.3389/fonc.2023.1155019 (PMC10189879; doi:10.3389/fonc.2023.1155019)
Supplement: Supplementary file 1 [file Table_1.docx]

Supplementary Material

Table S1. Treatment information of patients with metastatic gastrointestinal cancers.

| Cancer types | Treatment lines | No. of patients | Therapeutic schedule | | |
| --- | --- | --- | --- | --- | --- |
|  |  |  | ICIs | Targeted drug | Chemotherapeutic regimen |
| Esophageal cancer | 3 | 1 | Pembrolizumab | - | Paclitaxel |
|  | 1 | 1 | Nivolumab | Apatinib | - |
|  | 3 | 1 | Camrelizumab | - | Capecitabine |
|  | 3 | 1 | Sintilimab | - | Paclitaxel |
|  | 1 | 1 | Camrelizumab | Apatinib | - |
|  | 1 | 18 | Camrelizumab | - | Paclitaxel+Nedaplatin/Cisplatin |
|  | 1 | 16 | Sintilimab | - | Paclitaxel+Nedaplatin/Oxaliplatin/Lobaplatin |
|  | 2 | 1 | Pembrolizumab | - | Paclitaxel |
|  | 1 | 1 | Camrelizumab | - | S-1 |
|  | 1 | 2 | Camrelizumab | - | Paclitaxel |
|  | 3 | 3 | Camrelizumab | - | Paclitaxel |
|  | 3 | 1 | Camrelizumab | Apatinib | - |
|  | 1 | 1 | Sintilimab | - | Irinotecan |
|  | 2 | 2 | Camrelizumab | - | Paclitaxel |
|  | 1 | 1 | Sintilimab | - | S-1+Lobaplatin |
|  | 2 | 1 | Camrelizumab | Apatinib | - |
|  | 3 | 1 | Sintilimab | - | Etoposide+Cisplatin |
|  | 1 | 1 | Camrelizumab | - | Etoposide+Cisplatin |
|  | 1 | 1 | Sintilimab | - | S-1 |
|  | 3 | 1 | Camrelizumab | - | Paclitaxel+Cisplatin |
|  | 2 | 2 | Sintilimab | - | Paclitaxel+Nedaplatin/Oxaliplatin |
|  | 1 | 1 | Camrelizumab | Apatinib | Paclitaxel |
|  | 1 | 1 | Toripalimab | Surufatinib | - |
|  | 1 | 1 | Tislelizumab | - | Paclitaxel |
|  | 2 | 2 | Camrelizumab | - | Paclitaxel+Cisplatin/Oxaliplatin |
|  | 1 | 4 | Pembrolizumab | - | Paclitaxel+Cisplatin/Carboplatin |
|  | 1 | 2 | Tislelizumab | - | Paclitaxel+Cisplatin |
|  | 1 | 2 | Camrelizumab | Apatinib | Paclitaxel+Cisplatin |
|  | 1 | 1 | Pembrolizumab | - | Paclitaxel |
|  | 1 | 1 | Sintilimab | - | Paclitaxel+Capecitabine |
|  | 2 | 1 | Pembrolizumab | - | Paclitaxel+Carboplatin |
|  | 3 | 1 | Sintilimab | Trastuzumab | - |
|  | 1 | 2 | Sintilimab | - | Cisplatin+Fluorouracil |
|  | 2 | 1 | Camrelizumab | - | Irinotecan+Capecitabine |
|  | 2 | 1 | Camrelizumab | Anlotinib | - |
| Gastric cancer | 2 | 2 | Pembrolizumab | Lenvatinib | - |
|  | 1 | 8 | Camrelizumab | - | Oxaliplatin+Capecitabine/S-1 |
|  | 3 | 1 | Sintilimab | Apatinib | - |
|  | 3 | 5 | Camrelizumab | Apatinib | - |
|  | 3 | 1 | Toripalimab | Apatinib | - |
|  | 3 | 2 | Pembrolizumab | Apatinib | - |
|  | 2 | 5 | Camrelizumab | Apatinib | - |
|  | 4 | 1 | Pembrolizumab | Trastuzumab | Paclitaxel |
|  | 3 | 1 | Tislelizumab | - | Capecitabine |
|  | 2 | 10 | Nivolumab | - | Paclitaxel+S-1 |
|  | 4 | 2 | Toripalimab | Apatinib | - |
|  | 3 | 2 | Tislelizumab | Apatinib | - |
|  | 3 | 5 | Camrelizumab | Apatinib | Paclitaxel |
|  | 1 | 3 | Sintilimab | Trastuzumab | Oxaliplatin/Lobaplatin+S-1 |
|  | 3 | 1 | Sintilimab | - | Oxaliplatin+Capecitabine |
|  | 1 | 11 | Sintilimab | - | Oxaliplatin+Capecitabine/S-1 |
|  | 2 | 1 | Camrelizumab | Apatinib | Paclitaxel |
|  | 3 | 1 | Sintilimab | Apatinib | Irinotecan |
|  | 1 | 4 | Camrelizumab | - | Paclitaxel+Oxaliplatin |
|  | 2 | 1 | Sintilimab | Apatinib | Oxaliplatin |
|  | 3 | 1 | Toripalimab | - | S-1 |
|  | 3 | 1 | Tislelizumab | Trastuzumab | Irinotecan+Capecitabine |
|  | 1 | 1 | Tislelizumab | Apatinib | - |
|  | 3 | 1 | Tislelizumab | - | Irinotecan+Capecitabine |
|  | 3 | 3 | Sintilimab | - | Paclitaxel+S-1 |
|  | 1 | 1 | Tislelizumab | Trastuzumab | - |
|  | 3 | 2 | Camrelizumab | Trastuzumab | Irinotecan |
|  | 3 | 1 | Camrelizumab | Apatinib | S-1 |
|  | 2 | 2 | Camrelizumab | Trastuzumab | Paclitaxel |
|  | 3 | 3 | Tislelizumab | Apatinib | Irinotecan |
|  | 1 | 1 | Tislelizumab | - | Paclitaxel |
|  | 1 | 4 | Camrelizumab | - | S-1 |
|  | 2 | 1 | Sintilimab | - | Oxaliplatin+S-1 |
|  | 1 | 1 | Tislelizumab | - | Paclitaxel+S-1 |
|  | 1 | 1 | Camrelizumab | - | Paclitaxel+Oxaliplatin+S-1 |
|  | 1 | 1 | Tislelizumab | Apatinib | Oxaliplatin+S-1 |
|  | 1 | 1 | Pembrolizumab | Lenvatinib | - |
|  | 2 | 2 | Tislelizumab | Apatinib | Irinotecan |
|  | 2 | 1 | Tislelizumab | Trastuzumab | Paclitaxel+Capecitabine |
|  | 2 | 1 | Nivolumab | - | Paclitaxel |
|  | 1 | 1 | Sintilimab | Apatinib | Paclitaxel+S-1 |
|  | 1 | 1 | Tislelizumab | Trastuzumab | S-1 |
|  | 2 | 1 | Tislelizumab | Apatinib | Paclitaxel+S-1 |
|  | 3 | 1 | Tislelizumab | Apatinib | Cisplatin |
|  | 2 | 1 | Camrelizumab | Trastuzumab | Oxaliplatin+S-1 |
|  | 2 | 1 | Sintilimab | - | Paclitaxel+Oxaliplatin |
|  | 3 | 1 | Camrelizumab | - | Irinotecan |
|  | 3 | 1 | Pembrolizumab | Trastuzumab | Oxaliplatin+Capecitabine |
|  | 2 | 1 | Tislelizumab | - | Irinotecan+Capecitabine |
| Hepatocellular cancer | 2 | 1 | Pembrolizumab | Lenvatinib | - |
|  | 2 | 1 | Sintilimab | Sorafenib | - |
|  | 1 | 10 | Camrelizumab | Lenvatinib | - |
|  | 2 | 3 | Sintilimab | Lenvatinib | - |
|  | 1 | 5 | Tislelizumab | Lenvatinib | - |
|  | 1 | 5 | Camrelizumab | Apatinib | - |
|  | 2 | 1 | Toripalimab | Lenvatinib | - |
|  | 2 | 1 | Camrelizumab | Sorafenib | - |
|  | 1 | 9 | Pembrolizumab | Lenvatinib | - |
|  | 1 | 1 | Sintilimab | Sorafenib | - |
|  | 2 | 1 | Sintilimab | Regorafenib | - |
|  | 2 | 3 | Camrelizumab | Apatinib | - |
|  | 2 | 1 | Camrelizumab | Bevacizumab | - |
|  | 2 | 1 | Tislelizumab | Lenvatinib | - |
|  | 1 | 1 | Sintilimab | Bevacizumab | - |
|  | 1 | 1 | Camrelizumab | Sorafenib | - |
|  | 3 | 1 | Camrelizumab | Apatinib | - |
|  | 1 | 4 | Sintilimab | Lenvatinib | - |
|  | 2 | 1 | Camrelizumab | Lenvatinib | - |
| Biliary tract cancers | 1 | 2 | Toripalimab | Lenvatinib | - |
|  | 1 | 1 | Sintilimab | Lenvatinib | - |
|  | 2 | 1 | Camrelizumab | - | Paclitaxel+S-1 |
|  | 1 | 2 | Pembrolizumab | Lenvatinib | - |
|  | 1 | 1 | Sintilimab | Lenvatinib | Paclitaxel+S-1 |
|  | 1 | 3 | Camrelizumab | Lenvatinib | - |
|  | 2 | 2 | Pembrolizumab | Lenvatinib | - |
|  | 2 | 1 | Camrelizumab | Lenvatinib | - |
|  | 2 | 2 | Sintilimab | Lenvatinib | - |
|  | 1 | 2 | Camrelizumab | - | Paclitaxel+S-1 |
|  | 3 | 1 | Camrelizumab | - | Irinotecan+Capecitabine |
